# Gingival recession in school kids aged 10-15 years in Udaipur, India

**DOI:** 10.4103/0972-124X.51889

**Published:** 2009

**Authors:** Anmol Mathur, Manish Jain, Koushal Jain, Mahima Samar, Balasubramanya Goutham, Prabu Durai Swamy, Suhas Kulkarni

**Affiliations:** *Department of Preventive and Community Dentistry, Darshan Dental College and Hospital Udaipur, Rajasthan – 313 001, India*

**Keywords:** Attached gingiva, children, epidemiology, gingival recession, risk factors

## Abstract

**Aim:**

The study aimed to determine the incidence of gingival recession in the mandibular central incisor region among school children aged 10-15 years in Udaipur (India).

**Materials and Methods:**

A sample of 1800 males and female kids were examined in a mobile dental unit. World Health Organisation (WHO) rules and standards were followed.

**Result:**

Gingival recession, when compared, with respect to age, mean clinical crown length, anterior crowding and frenal involvement was significant (p less than 0.00) with respect to affected teeth.

## INTRODUCTION

Localized gingival recession occasionally presents a problem in children and there is some confusion regarding the etiology and pathogenesis of such defects. Sognnaes RF[1] found a significant relationship between gingival recession and various factors like faulty tooth brushing technique (gingival abrasion), wrong positioning of tooth, friction from soft tissue (gingival ablation), gingival inflammation and high frenum attachment. Trauma from occlusion has also been suggested, but its mechanism of action has never been demonstrated. Orthodontic tooth movement in a labial direction has been shown in monkeys to result in loss of marginal bone and connective tissue attachment, as well as in gingival recession.[2]

Baker and Seymour[3] have also suggested that plaque induced inflammation is responsible for gingival recession. Stoner and Masdyasna[4] found no association between calculus and gingival recession but found that it was closely related to the width of keratinized gingiva. Jukka *et al*.[5] found the prevalence of gingival recession more common among the girls in the earlier age group and equally among both with age.

Woofter[6] assumes that recession may be a physiological process related to aging. There is some doubt regarding diagnosis of gingival recession[7] with certainty, before 12 years of age, and it has been suggested that apparent recession in the younger children was due to more delay in the maturation of the gingivae of adjacent paired tooth than to true recession of the gingivae of the apparently affected tooth. However, convincing evidence for a physiological shift[8] of the gingival attachment has never been presented.

Fermin A. Carranza[9] concluded that the gradual apical shift is most probably the result of the cumulative effect of minor pathologic involvement and/or repeated minor direct trauma to the gingiva.

### Clinical Significance

Several aspects of gingival recession make it clinically significant. Exposed root surfaces are susceptible to caries. Wearing away of the cementum exposed by recession leaves an underlying dentinal surface which is extremely sensitive, particularly to the touch. Hyperemia of the pulp and associated symptoms may also result from exposure of the root surface.[10] Interproximal recession creates space in which plaque, food and bacteria can accumulate.

## MATERIALS AND METHODS

The incidence of gingival recession in the mandibular central incisor region was examined using the stratified random sampling technique. A sample of 1800 males and females were selected from five zones in Udaipur, for a fair sample selection. Schools from each zone: North, South, East, West and Center were randomly selected, equal number of boys and girls were chosen from each school. At the onset, informed consent for data collection was obtained from the Principals of the school and parents of the individuals who participated in the study. No such study has taken place in this part of the country till date. The period for data collection was from November 6, 2007 to March 29, 2008. A dental examination was carried out in a mobile dental unit. “Interchangeable plane mouth mirrors and Williams periodontal probe no.0” was used to measure the clinical crown length of the affected and adjacent teeth. The following information was recorded:

Clinical crown length was measured for affected and adjacent teeth, using Williams's periodontal probe. Measurements were made at the labial midline from the gingival crest to the incisal surface. Gingival inflammation was recorded on the labial aspect of mandibular incisor using the gingival index of Loe and Sillness.[11]

For anterior crowding, the position of each mandibular central incisor was classified according its relation to the regular curve of the arch as described by Stoner and Mazdyasma[12] where 0=correctly positioned or instanding, 1=when the tooth was labially placed or absent.

Frenal involvement in the affected and adjacent teeth was recorded according to the classification of Powell and Mc Entery.[13] Accodingly, 0= No Frenal involvement, 1= Frenal insertion close to the gingival margin but no retraction of gingiva, 2= Narrow Frenal insertion with retraction of gingiva, 3= Broad Frenal insertion with retction of gingiva.

In data collection, methods and standards recommended by WHO, 1997, have been followed. The response rate to this study was 82%. Other relevant information was also recorded. The Ethical clearance for this study was taken from the ethical committee of “Darshan Dental College”.

Exclusion criteria: Of the 1800 students, 324 had gingival recession in the mandibular central incisor region. The remaining 1236 students were excluded as they did not have gingival recession; 172 were absent or not co-operating, 68 did not have mandibular central incisors.

The agreement (Kappa Statistics) for diagnosis of gingival recession and there supporting criterias was determined (Field Team v/s Expert) and inter examiner variability by a group of four people is 91.2% 2 days prior to the examination. Statistical analysis was carried out using Chi-squared test for the significance of apparent association between Recession and Recorded variables.

## RESULTS

Gingival recession was observed in 324 (18%) of 1800 male and female pupils.

**Graph 1:**
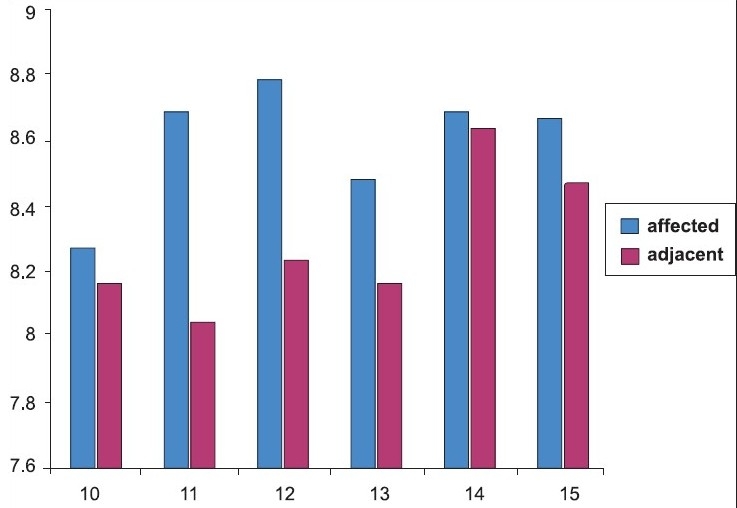
Showing mean difference of crown length between affected and adjacent teeth of age group ranging 10-15 years

Table 1 and Graph 1 shows the mean and standard deviation of clinical crown length of the affected and adjacent lower central incisor teeth by age groups. There was a significant difference in the clinical crown length of teeth with gingival recession between the various age groups (*P*<.000).

**Table 1:** Mean and standard deviation of clinical crown length, affected and adjacent mandibular central incisor teeth by age group

Age	Students	Affected X‘± SD*	Adjacent X ± SD
10	38	8.29 ±‘0.89	8.18‘± 1.09
11	34	8.71‘±0.84	8.06‘± 1.10
12	48	8.81 ±‘0.97	8.25‘± 1.02
13	60	8.50 ±‘1.13	8.18‘± 1.13
14	70	8.71 ± 0.97	8.66‘± 1.01
15	74	8.69 ± 1.02	8.49‘± 0.86

X± SD = Mean standard deviation; t = 3.522, *P* = 0.000 (by ‘t’ test)

**Graph 2:**
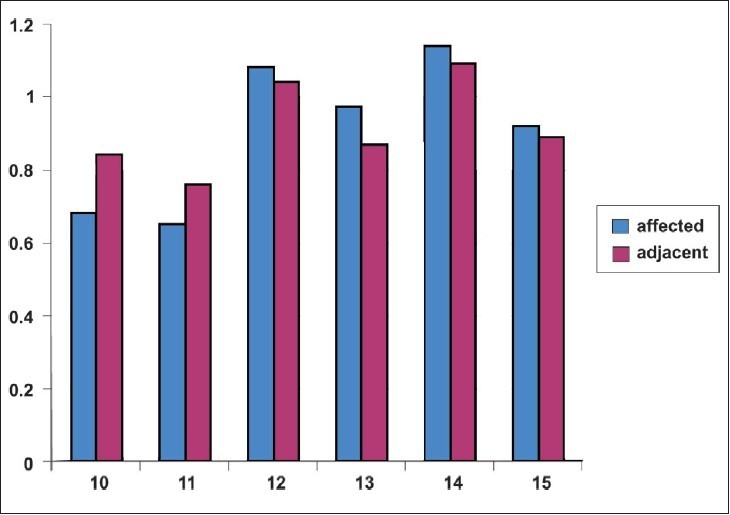
Showing mean difference of gingival inflammation between affected and adjacent teeth of age group ranging from 10-15 years

Table 2 and Graph 2 shows that gingival inflammation was less frequent on teeth with gingival recession than for the adjacent teeth without recession (x^2^=1.133(a), df 3, p=.769).

**Table 2:** Association of gingival inflammation, anterior crowding and frenal involvement with affected and adjacent tooth

Variable score	Affected tooth		Adjacent tooth		Chi-square
			
	n	%	n	%	
Gingival 0	88	27.2	84	25.9	C.S=1.133
Inflamm 1	174	53.7	186	57.4	DF=3
2	54	16.7	46	14.2	*P*=0.769
3	8	2.5	8	2.5	-
Anterior					C.S=21.920
Crowdin 0	282	87	234	72.2	DF=1
1	42	13	90	27.8	*P*=0.000
Frenal 0	244	75.3	278	85.8	C.S=18.46
INVOL. 1	68	21	46	14.2	DF=2
2	12	3.7	0	0	*P*=0.000

**Graph 3:**
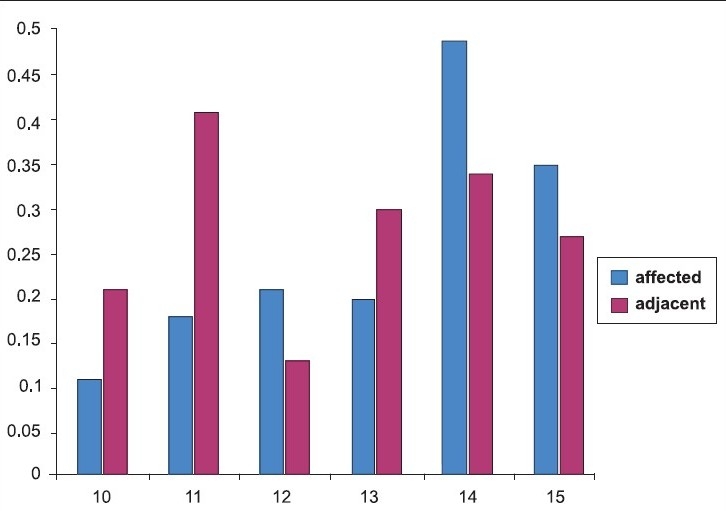
Showing mean difference of arch relationship between affected and adjacent teeth of age group ranging from 10-15 years

Data collected for all pupils were statistically analyzed and revealed significant differences in gingival recession by age (x^2^=89.665, df=15, *P*=.000) [Table 3 and Graph 3].

**Table 3:** Distribution of gingival inflammation, anterior crowding and frenal involvement by age

Age	Inflammation score						Anterior crowding score				Frenal involvement score					
			
	0		1		2,3		0		1		0		1		2,3	
								
	N	%	N	%	N	%	N	%	N	%	N	%	N	%	N	%
10	30	17.44	36	10	10	8.62	64	12.40	12	9.09	68	13.02	8	7.01	0	0
11	24	13.95	40	11.11	4	3.44	48	9.30	20	15.15	64	12.26	4	3.50	0	0
12	14	8.13	62	17.22	20	17.24	80	15.50	16	12.12	76	14.55	20	17.54	0	0
13	28	16.27	74	20.55	18	15.51	90	17.44	30	22.72	102	19.54	16	14.03	2	16.66
14	32	18.60	62	17.22	46	39.65	112	21.70	28	21.21	88	16.85	48	42.10	4	33.33
15	44	25.58	86	23.88	18	15.51	122	23.64	26	19.69	124	23.75	18	15.78	6	50
Chi-square		X^2^=89.665, df=10, *P*=0.000					X^2^=7.536, df=5, *P*=0.184					X^2^=51.286, df=10, *P*=0.000				

Gingival score of 1 was most frequent in relation to the adjacent teeth and formed 57.4% of the total no. of adjacent teeth. While the gingival score of 3 is equal in both affected and adjacent teeth constituting 2.5% of the total of there respective teeth.

There was a significant correlation between the teeth in the dental arch and the occurrence of gingival recession (x^2^=21.920, df=1, *P*=.000). No significant difference in tooth position of teeth with gingival recession by age was observed (x^2^=7.536, df=5, *P*=.184)

A significant association between frenal involvement and gingival recession was found (x^2^=18.460(a), df=2, *P*=.000). The majority of the affected and adjacent teeth (80.6%) were not affected by frenal insertion, however narrow frenal insertion was not found in relation to the adjacent teeth. Broad frenal insertion was completely absent during the study.

There was also a significant changes in frenal involvement on teeth with gingival recession by age (x^2^=51.286(a), df=10, *P*=.000).

### Other relevant information

Calculus deposits were not related to the occurrence of gingival recession and were present in 68 children. Recent Apthous Ulceration was noted in 23 cases. Thin, almost transparent tissue was observed over the unaffected adjacent teeth in a number of subjects and is presumably associated with active soft tissue remodeling. Several students seemed concerned to prevent the gingival recession. Among them, 52 students have gone through the reparative treatment and proper plaque control programme. This study shows that rural school students are more prone to recession compared to students in urban areas. It was also seen that more number of boys than girls had recession in each respective age groups.

## DISCUSSION

The results of this study show that the higher percentage of teeth with gingival recession is frequently associated with clinical crown length, arch relationship and frenum involvement while gingival inflammation is less significant. It appears from this study that gingival recession of mandibular central incisor at age 10-15 years is less affected by gingival inflammation (p greater than 0.76) but with the increase in age gingival inflammation get significant (p less than 0.00). We can prove our result with the help of different study models about, effects of aging on progression of periodontal disease. A comparison of developing gingivitis between young and older individuals demonstrated a greater inflammatory response in older subjects.[14–17] This effect must be because older individuals are more prone to poor oral hygiene habits like tobacco chewing and cigarette smoking.

Our observation is in partial accordance with the results presented by Sognnaes RF[1] as he found a significant relationship between gingival recession and various factors like faulty tooth brushing technique, tooth malposition, gingival ablation and high frenal attachment which is in accordance to our results but it also included gingival inflammation as a major criteria for gingival recession which is in partial acceptance with this study.

According to our study, gingival recession of mandibular central incisor can manifest itself as early as 10 years of age which is in accordance with Powell and Mcenetry[13] and Parfitt and Major.[18] when tested in the similar age groups with this study.

Our observation is in agreement with the results presented by Volchansky and Jones[7] which demonstrated that gingival height did not stabilize in the central incisor region before the age of 12 years. In our study we mentioned that gingival height did not stabilize i.e leading to recession in earlier age group (10-15 years).

HOLLIST[19] reported that children usually started to use the chewing stick (Miswak) as a traditional habit between the age of seven and 10 year and he also reported that unsuitably varying length of miswak will easily traumatize the periodontia leading to recession in earlier age group in accordance with us. We noticed recession of quite dramatic proportions even in the six year age group well before the eruption of lateral incisors, suggesting that even where crowding might become complicating factor at a later date, the disuse destruction was already established prior to the manifestation of crowding.

A study of Salwa and Mohammad Farouk[20] recorded a prevalence of gingival recession in the mandibular central incisor region of 9.88% in a group of 1336 children aged 10-15 years. As per this study the prevalence rate of gingival recession increases to 18% in a group of 1800 children having age 10-15 years, it means the trend of gingival recession has been increased in the same age group.

In this study tooth malposition is one of the major criteria for gingival recession. Similar type of study was done by Anna Andlin Sobocki.[21] He examined 38 children aged 7-12 years with a gap of two years to determine whether facial/lingual tooth position changes were related to alteration of the width of attached and keratinized gingival and the clinical crown height. The result showed that significant alteration in the width of attached and keratinized gingiva took place when teeth changed position in labial or lingual direction. The changes in gingival widths could, to some extent be coupled to changes in clinical crown height. In teeth moving lingually gingival width increased and clinical crown height decreased. In teeth moving facially, the gingival width decreased, and the facial gingival sometimes receded. However, in our study, anterior crowding is more significant in relation to adjacent teeth, not having gingival recession.

Clinical crown height is an objective measure of the position of gingival margin which could be used in determining the “normal position” of the gingival margin according to Volchansky and Cleaton Jones,[7] the aim of this study was to do a systematic review of published clinical crown heights in the human permanent dentition to compare the measurements and to see if the clear trend with age exists. There is a statistically significant increase in clinical crown height of central and lateral incisors with age that slows down as age increases. Similarly, in our study significant increase in clinical crown height of central and lateral incisors with age is seen as the age increases, age is in significant association with gingival recession (*P*=0.00).

This study favours the results presented by Elifuraha *et al*.[22] that the risk factors for gingival recession were identified as male sex, lower educational status, presence of plaque and gingival inflammation, but did not match with the results of Cahen *et al*.,[23] as per whose result sex has a significant influence on caries indices but not on plaque, calculus or gingival indices.

The finding of Sobocki *et al.*[21] that gingival recession in mandibular incisors in young children often improves over time suggests that preventive or reparative treatment in this part of the developing dentition may not be necessary. Decisions regarding such treatment should be postponed until any spontaneous improvement has taken place.

According to the Raymer *et al*.,[24] the self assessment approaches can be effective in improving the long term periodontal health status of teenagers. As with much of dental epidemiology the value of cross sectional surveys in the study of gingival recession is limited. Longitudnal studies are required so that effects of resolution of gingival inflammation and of relief of crowding may be followed under conditions of a controlled clinical trial. Only in this way the complex instructions influencing gingival recession may be unraveled.
